# Consumption and Climate

**Published:** 1871-09

**Authors:** 


### CONSUMPTION AND CLIMATE.

Some years ago, it was said that very few persons died of consumption in California. This gave rise to the statement that the climate was quite favorable for lung affections, leaving out of the account that it was a new country, and also that the means of knowing the number who perished of that disease were so very imperfect that it was altogether impossible to arrive at any intelligent conclusion on the subject. To-day, no one thinks of going to the Golden State to get rid of lung disease.

Within a very short time Minnesota has been the Mecca of the doomed, with the result that two thirds of those who visit that State with consumptive disease perish by it. To benefit a consumptive really and radically, the climate must be cool, dry, still; cool, to invigorate the appetite and digestion; dry, to prevent the too rapid evaporation by the dampness; still, that heat may not be so rapidly carried from the body as to induce chills, fevers, and general wasting away. Ten or fifteen years ago the climate might have been more favorable than now, but the face of the country has been changed in the progress of civilization, and with it the conditions of health and disease. It is only in the cooler parts of that State that any advantages are claimed for consumptives. But the spring time is most miserably damp, raw, and chilly; so much so, indeed, that the better classes who can afford it, and who are at all invalids, escape from its pernicious influences. In the winter the thermometer goes as far down as forty degrees below zero, and often with so cutting a wind, that the healthiest and sturdiest people find it impossible at times to be out longer than half an hour at once. For some weeks in the fall the air is propitious for the invalid; but the long, bleak, drear winter is death, because the ailing are cooped up in warm rooms for weeks and months together. A careful and dispassionate observer, himself an invalid, estimates that only from five to ten per cent. of those in the early stages of decided consumption ever get well by going to Minnesota. The winters are not so severe as in New England; there is not so much snow, and the rivers are navigable for a greater portion of the year than the Erie Canal in New York State, yet the average temperature is much colder than in Vermont, and on days not a few, no sane person thinks of going out of doors, except but for the fewest number of visits. The average rain-fall is thirty-seven inches for a few years past; hence the dampness must be great, the thaws distressing, and the changes frequent, sudden, and severe. In the treatment of some thousands of cases of actual or feared consumption in the last third of a century, it is the editor's conviction that any one who has actual consumption will live longer by staying at home, in whatever latitude that home may be, if it is a home of his own; for between hired attentions and those which love and affection command, there can be no comparison, the latter having hygienic advantages which are of incalculable benefit.

Since writing the above, an authoritative announcement has been made, strikingly confirming the statements made. The Secretary of the Young Men's Christian Association at St. Paul, writes to the "Watchman" newspaper, saying that his society, during the last year, has spent a large sum in nursing and in burying Christian young men who came to St. Paul in pursuit of health, only to die."

We have long considered it one of the inhumanities of man to man, in so glibly advising persons to go from home to distant places, involving, many times, ruinous expenses, especially when it is considered as a last resort — advice often given, when everything possible has been done and tried without efficacy, merely on the ground that possibly it might make some change for the better, while the overshadowing probabilities are that death will be the result anyhow. Any man who is considered by an intelligent physician to have actual consumption, ought by all means to stay at home.

